# Extrapulmonary onset manifestations of COVID-19

**DOI:** 10.6061/clinics/2021/e2900

**Published:** 2021-06-29

**Authors:** Josef Finsterer, Fulvio A. Scorza, Carla A. Scorza, Ana C. Fiorini

**Affiliations:** I Klinik Landstrasse, Messerli Institute, Vienna, Austria.; II Disciplina de Neurociencia, Escola Paulista de Medicina/Universidade Federal de Sao Paulo (EPM/UNIFESP), Sao Paulo, SP, BR.; III Programa de Estudos Pos-Graduados em Fonoaudiologia, Pontificia Universidade Catolica de Sao Paulo (PUC-SP), Sao Paulo, SP, BR, Departamento de Fonoaudiologia, Escola Paulista de Medicina/Universidade Federal de Sao Paulo (EPM/UNIFESP), Sao Paulo, SP, BR.

**Keywords:** Clinical Presentation, COVID-19, Onset, SARS-CoV-2, Variability

## Abstract

Coronavirus disease (COVID-19) usually starts with pulmonary signs and symptoms. However, in some cases, the initial clinical presentations are extrapulmonary. This literature review aimed at summarizing and discussing the extrapulmonary onset manifestations of COVID-19. The most frequent initial extrapulmonary manifestations include hypogeusia, hyposmia, non-specific abdominal symptoms, corneal congestion, and deep venous thrombosis. Several rarer extrapulmonary manifestations in locations such as the brain, peripheral nerves, muscles, eyes, ears, myocardium, intestines, skin, or vessels have been additionally reported as onset presentations of COVID-19. In conclusion, it is crucial for clinicians and health care providers to consider extrapulmonary presentations at the onset of COVID-19 to avoid overlooking the infection and contributing to the spread of the disease.

## INTRODUCTION

The most common initial manifestations of severe acute respiratory syndrome coronavirus 2 (SARS-CoV-2) infection are fever, fatigue, myalgia, breathlessness, and cough due to the lungs being affected (1). However, coronavirus disease (COVID-19), the disease manifestation of SARS-CoV-2 infection, may also initially manifest as extrapulmonary abnormalities (2). This review aimed at summarizing and discussing the extrapulmonary initial manifestations of COVID-19.

## MATERIALS AND METHODS

We retrieved publications that met the inclusion criteria from the PubMed and Google Scholar databases after applying appropriate search terms (COVID-19, SARS-CoV-2, onset, initial, clinical presentation, extrapulmonary, central nervous system, neurological, cardiac, myocardial, arrhythmias, gastrointestinal, renal, liver, pancreas). We also searched the reference lists of these publications for appropriate articles. The search and analysis were restricted to publications in 2020.

### Ethical approval

We did not perform any experimental studies with human participants or animals for this literature review. Thus, this study was exempt from ethical approval.

## RESULTS

**Figure 1:**
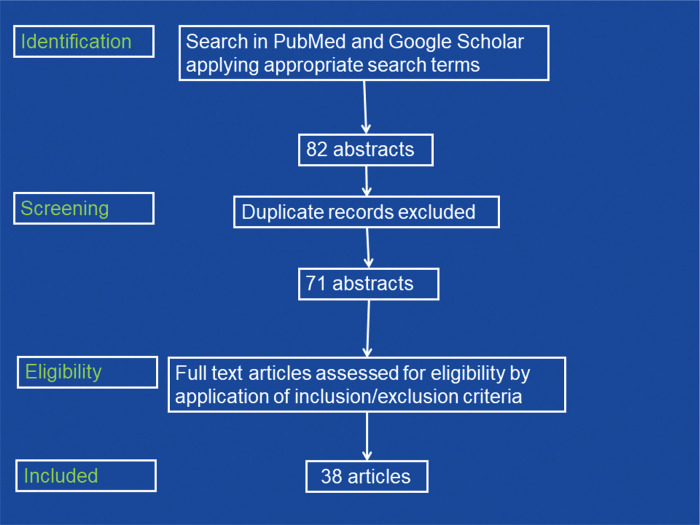
Flowchart of the study selection process.

A total of 38 articles were included in this review (Figure 1). In the majority of the included cases, extrapulmonary initial manifestations were described in case reports or series. Organs or tissues in which COVID-19 may initially clinically manifest include the central nervous system (CNS), peripheral nervous system, skeletal muscles, eyes, ears, heart, intestines, vessels, skin, blood, or other compartments (Table 1). The most frequent extrapulmonary initial manifestations of COVID-19 are hyposmia, hypogeusia, non-specific abdominal symptoms, corneal congestion, and deep venous thrombosis (Table 1). Reported initial CNS manifestations of COVID-19 include meningitis, seizures, stroke, intracerebral bleeding, headache, delirium, cognitive impairment, myelitis, and acute, disseminated encephalomyelitis (Table 1) (3). Initial extrapulmonary COVID-19 manifestations of the peripheral nerves include facial nerve palsy or dysautonomia (Table 1) (4). The muscle can be the first site affected by SARS-CoV-2 in the form of myositis or rhabdomyolysis (Table 1). Initial manifestations of COVID-19 in the eyes include conjunctival congestion, conjunctivitis, panuveitis, or optic neuritis. One patient reportedly had an initial COVID-19 manifestation of sensorineural hearing loss (Table 1) (5). Myocarditis was also the first clinical manifestation of the infection in another patient (Table 1) (6). Intestinal initial manifestations of COVID-19 include abdominal pain, diarrhea, or vomiting (Table 1) (7). More rarely, gastrointestinal bleeding, mesenteric adenopathy (enlarged mesenteric lymph nodes), or pancreatitis has been reported at the onset of the infection (Table 1). Patients with COVID-19 are prone to venous thrombosis, most likely due to hypercoagulability. However, only deep venous thrombosis has been reported as an initial COVID-19 manifestation (8). In two patients, arterial thrombosis was the initial manifestation of COVID-19 (Table 1) (8). In a patient with juvenile ischemic stroke, acute occlusion of the common carotid artery was suspected as the first manifestation of COVID-19 (9). Interestingly, maculopapular rash and chilblain (frostbite)-like lesions have been also reported as initial clinical manifestations of the infection. In a newborn patient, COVID-19 manifested initially with neonatal apnea (Table 1) (10). In a review of 25 COVID-19 patients, orofacial manifestations (ulcer, vesicular-bullous or macular lesions, and sialadenitis) were the first signs of the disease in four cases (11). A limitation of the study was that some articles might have been missed because of the selection of the search terms.

## DISCUSSION

This review shows that various extrapulmonary manifestations may characterize the initial appearance of the infection. The most common extrapulmonary presentations at disease onset include hypogeusia, hyposmia, diarrhea, vomiting, corneal congestion, and thrombosis. Considering the systemic nature of the infection is crucial as it may strongly determine the therapy course and outcome of the individual patient. Investigations that can confirm extrapulmonary manifestations of COVID-19 in cases of clinical suspicion include cerebral imaging, electroencephalography, electrocardiogram, echocardiography, blood tests (creatine-kinase, troponin, pro brain-type natriuretic peptide, renal function parameters, amylase, lipase, and liver function parameters), abdominal computed tomography, nerve conduction studies, needle electromyography, and cerebrospinal fluid investigations.

The reason why COVID-19 can manifest initially in areas other than the lungs is unknown, but it may be due to exposure of the eyes and skin to aerosols, due to ingestion of the virus, due to distribution of the virus via the blood stream, or due to retrograde transport of the virus along peripheral nerves to the CNS. It is also conceivable that patients remain asymptomatic at initial viral exposure of the lungs because of a low virus concentration, but the virus spreads via the blood stream from the lungs to other organs and replicates, causing the patient to present symptoms in those organs. It is also imaginable that clinical pulmonary manifestations are initially suppressed because of an intact immune response that later becomes increasingly compromised because of the spread and generalization of the disease, resulting in clinical manifestations in extrapulmonary locations with a high viral load (12). Although extrapulmonary manifestations of COVID-19 may be misleading with regard to the suspicion of COVID-19, clinicians should consider that the infection may start with atypical presentations and that the conditions described in this review can suggest COVID-19.

## CONCLUSIONS

Overall, there is evidence that COVID-19 occasionally has initial clinical manifestations in areas other than the lungs. Extrapulmonary organs that are most frequently initially affected are the intestines. Health care systems should adapt their strategies and management of the infection and of patients with clinical manifestations accordingly. Classical pulmonary manifestations may be absent; nonetheless, patients may have COVID-19 even in the absence of fever.

## RECOMMENDATIONS

There is a need to alert attending physicians that COVID-19 may start with unusual presentations. Attending doctors should maintain a high index of suspicion for systemic manifestations of COVID-19.

## AUTHOR CONTRIBUTIONS

All of the authors qualify for authorship and have checked the manuscript for plagiarism. Finsterer J conceived and designed the study, conducted the research, provided research materials, collected and organized data, and wrote the initial and final manuscript drafts. Scorza FA contributed to the conception and design, analyzed and interpreted data, provided logistic support, contributed to the manuscript drafting, revision, literature search, critically reviewed the manuscript and approved its final version. Scorza CA contributed to the conception and design, analyzed and interpreted data, provided logistic support, contributed to the manuscript drafting, literature search, and approval of the final version of the manuscript. Fiorini AC contributed to the conception and design, analyzed and interpreted data, provided logistic support, contributed to the manuscript drafting and critical review, literature search, and approval of the final version of the manuscript.

## Figures and Tables

**Table 1:** Overview of the initial clinical manifestations of SARS-CoV-2-infected patients.

Organ	Initial manifestation	Frequency	Reference
CNS	Meningitis	rare	[Naz et al. (13)]
	Delirium	rare	[Poloni et al. (16)]
	ADEM	rare	[Abdi et al. (14)]
	Epilepsy	rare	[Pinna et al. (15)]
	Headache	rare	[Pinna et al. (15)]
	Ischemic stroke	rare	[Pinna et al. (15)]
	Intracerebral bleeding	rare	[Pinna et al. (15)]
	Short term memory impairment	rare	[Pinna et al. (15)]
	Transverse myelitis	rare	[Chakraborty et al. (17)]
	Altered mental state	rare	[Pinna et al. (15)]
	Confusion	rare	[Alkeridy et al. (18)]
	Acute encephalopathy	rare	[Farhadian et al. (19)]
Nerves	Hyposmia	frequent	[Ramasamy et al. (4)]
			[Chi et al. (20)]
	Hypogeusia	frequent	[Ramasamy et al. (4)]
			[Chi et al. (20)]
	Guillain-Barre syndrome	rare	[Zhao et al. (21)]
	Dysautonomia	rare	[Pinna et al. (15)]
	Facial palsy	rare	[Pinna et al. (15)]
Muscle	Myositis	rare	[Almadani et al. (22)]
	Rhabdomyolysis	rare	[Anklesaria et al. (23)]
			[Suwanwongse et al. (24)]
Eyes	Pan-uveitis	rare	[Benito-Pascual et al. (25)]
	Optic neuritis	rare	[Benito-Pascual et al. (25)]
	Conjunctivitis	rare	[Ozturker (26)]
	Conjunctival congestion	rare	[Chen et al. (27)]
	Retro-orbital pain	rare	[Ruiy et al. (28)]
Ears	Sensorineural hearing loss	rare	[Kilic et al. (5)]
Heart	Myocarditis	rare	[Naneishvili et al. (6)]
Intestines	Abdominal pain, cramps, nausea	frequent	[Dietrich et al. (7)]
			[Wu et al. (29)]
			[Amaral et al. (30)]
			[Remes-Troche et al. (31)]
			[Khader et al. (32)]
	Hepatopathy	frequent	[Mao et al. (33)]
	Mesenteric adenopathy	rare	[Noda et al. (34)]
	Gastrointestinal bleeding	rare	[Gulen and Satar (35)]
	Pancreatitis	rare	[Wang et al. (36)]
	Diarrhea	rare	[Yang et al. (37)]
Vessels	Deep venous thrombosis	frequent	[Erdinc et al. (8)]
	Artery thrombosis	rare	[Thompson et al. (38)]
			[Shao et al. (39)]
	Common carotid artery occlusion	rare	[Alkhaibary et al. (9)]
Skin	Chilblain-like lesions, bullae	rare	[Rubin et al. (40)]
	Maculopapular rash	rare	[Falkenhain et al. (41)]
Blood	Thrombocytopenia	rare	[Ahmed et al. (42)]
Other	Neonatal apnoea	rare	[Gonzalez-Brabin et al. (10)]
	Orofacial manifestations	rare	[Halboub et al. (11)]

SARS-CoV-2: severe acute respiratory syndrome coronavirus 2, ADEM: acute, disseminated encephalomyelitis, CNS: central nervous system, frequent: >9 cases reported, rare: <10 cases reported.
